# Neurocognitive Profiles of 22q11.2 and 16p11.2 Deletions and Duplications

**DOI:** 10.21203/rs.3.rs-3393845/v1

**Published:** 2023-12-29

**Authors:** Ruben Gur, Carrie Bearden, Sébastien Jacquemont, Khadije Jizi, Therese Amelsvoort van, Marianne van den Bree, Jacob Vorstman, Jonathan Sebat, Kosha Ruparel, Robert Gallagher, Ann Swillen, Emily McClellan, Lauren White, Terrence Crowley, Victoria Giunta, Leila Kushan, Kathleen O’Hora, Jente Verbesselt, Ans Vandensande, Claudia Vingerhoets, Mieke van Haelst, Jessica Hall, Janet Harwood, Samuel Chawner, Nishi Patel, Katrina Palad, Oanh Hong, James Guevara, Charles-Olivier Martin, Anne-Marie Bélanger, Stephen Scherer, Anne Bassett, Donna McDonald-McGinn, Raquel Gur

**Affiliations:** University of Pennsylvania; University of California at Los Angeles; Centre Hospitalier Universitaire Sainte-Justine; Maastricht University; Cardiff University; The Hospital for Sick Children; University of California, San Diego; University of Pennsylvania; University of California, Los Angeles; Department of Psychiatry and Psychology, Maastricht University; Cardiff University; MRC Centre for Neuropsychiatric Genetics and Genomics; Hospital for Sick Children; Centre for Addiction and Mental Health; Children’s Hospital of Philadelphia; University of Pennsylvania

## Abstract

Rare recurrent copy number variants (CNVs) at chromosomal loci 22q11.2 and 16p11.2 are among the most common rare genetic disorders associated with significant risk for neuropsychiatric disorders across the lifespan. Microdeletions and duplications in these loci are associated with neurocognitive deficits, yet there are few studies comparing these groups using the same measures. We address this gap in a prospective international collaboration applying the same computerized neurocognitive assessment. The Penn Computerized Neurocognitive Battery (CNB) was administered in a multi-site study on rare genomic disorders: 22q11.2 deletion (n = 397); 22q11.2 duplication (n = 77); 16p11.2 deletion (n = 94); and 16p11.2 duplication (n = 26). Domains examined include executive functions, episodic memory, complex cognition, social cognition, and sensori-motor speed. Accuracy and speed for each neurocognitive domain were included as dependent measures in a mixed-model repeated measures analysis, with locus (22q11.2, 16p11.2) and copy number (deletion/duplication) as grouping factors and neurocognitive domain as a repeated measures factor, with age and sex as covariates. We also examined correlation with IQ and site effects. We found that 22q11.2 deletions were associated with greater deficits in overall performance accuracy than 22q11.2 duplications, while 16p11.2 duplications were associated with greater deficits than 16p11.2 deletions. Duplications at both loci were associated with reduced speed. Performance profiles differed among the groups with particularly poor performance of 16p11.2 duplication on non-verbal reasoning and social cognition. Average accuracy on the CNB was moderately correlated with Full Scale IQ. No site effects were observed. Deletions and duplications of 22q11.2 and 16p11.2 have varied effects on neurocognition indicating locus specificity, with performance profiles differing among the groups. These profile differences can help inform mechanistic substrates to heterogeneity in presentation and outcome. Future studies could aim to link performance profiles to clinical features and brain function.

## INTRODUCTION

The “genetics first” approach has investigated recurrent rare CNVs, such as those associated with chromosome 22q11.2 and 16p11.2, providing evidence of increased risk for neurodevelopmental psychiatric disorders across the lifespan. This line of research builds on individuals diagnosed when presenting clinically for evaluation and care at health care facilities and centers that recruit for research on rare genetic disorders. There are common neurobehavioral features associated with these CNVs that manifest transdiagnostically in Attention Deficit Hyperactivity Disorder, Anxiety Disorders, Mood Disorders, Autism Spectrum Disorders, Schizophrenia and Psychosis Spectrum Disorders [1, 2]. Notably, features of neurodevelopmental psychiatric disorders associated with these CNVs are similar to the presentation and course of some idiopathic (behaviorally defined) neurodevelopmental disorders. Among rare CNVs, 22q11.2 deletion and duplication as well as 16p11.2 deletion and duplication have been examined for developmental psychiatric disorders including cognitive functioning. A survey conducted at the Geisinger Health System reported that 22q11.2 duplication (0.119%) and 16p11.2 deletion (0.078%) were the most prevalent CNVs and were associated with lifelong cognitive and psychiatric disabilities documented in electronic health records [2]. The extent and nature of neurocognitive deficits associated with these deletions and duplications varies, and studies to date have usually examined a single CNV or either deletions or duplications. Furthermore, these studies have used varied quantity and quality of neurocognitive assessments, with most focusing on an “intelligence quotient” (IQ) assessed as part of a clinical or research evaluation.

Notably, most studies on cognitive functioning in these CNVs were during childhood, adolescence or young adulthood (6–25 years). Less is known about cognitive functioning in adults. Investigations that examined only 22q11.2 deletion have documented a high prevalence of learning difficulties and intellectual disabilities (mostly mild-moderate) and a range of neurocognitive deficits [e.g., 3–7]. Longitudinal studies have suggested that these deficits are associated with psychiatric symptoms [8–12], and may drive their exacerbation [13]. These impairments comprise several neurobehavioral domains including executive functions and social functioning [3–15]. They are influenced by environmental factors [16] and are associated with abnormalities in brain maturation[17]. Fewer studies have examined 22q11.2 duplication only [18, 19] but these have likewise reported learning problems and cognitive deficits. In a study comparing deficits between 22q11.2 deletion and 22q11.2 duplication (n = 19 in each group), and another larger study (106 22q11.2 deletion, 38 22q11.2 duplication), it was concluded that patients with 22q11.2 duplication have a milder cognitive impairment than the 22q11.2 deletion counterparts [20].

Studies examining 16p11.2 deletion have likewise reported reduced intellectual functioning [21] and neurocognitive deficits partly associated with psychopathology [22–24]. There is also evidence for abnormalities in white matter integrity associated with these deficits [25]. Other studies have compared 16p11.2 deletion with 16p11.2 duplication [26–28] finding similar overall functioning across groups, although variance has been reported to be higher in 16p duplication^29^. In another study, the two groups (12 in each group) showed similar intellectual functioning [30]. A study comparing individuals with 16p11.2 deletion to 16p11.2 duplication on structural magnetic resonance imaging and neurocognitive performance (n = 79 in each group), reported distinct anatomic abnormalities associated with neurocognitive deficits [31]. A study of 82 individuals with 16p11.2 deletion, 50 with 16p11.2 duplication, 370 with 22q11.2 deletion, and 45 with 22q11.2 duplication reported that autism features were largely comparable across the four groups [32]. Significant variability in IQ was noted in CNVs of both loci.

The conclusions that can be drawn from studies to date are limited, as most were based on small sample sizes and because the extent and granularity of the neurobehavioral measures was variable. Because these CNVs are rare, attaining sufficient sample sizes of individuals for drawing firm conclusions requires multi-site collaborations with harmonized measures across sites. The Genes to Mental Health Network (G2MH) [1] was established for this purpose, to accrue a prospective sample with uniform assessment, implementation protocol, and shared data management and quality control. The present study reports the neurocognitive profile of these four groups (22q11.2 deletion/ duplication, 16p11.2p deletion/duplication) based on administration of the same neurocognitive battery across sites. The Penn Computerized Neurocognitive Battery (CNB) offers a neuroscience-based assessment of major behavioral domains linked to brain systems based on functional neuroimaging [11, 33–35]. It provides measures of executive functions, episodic memory, complex cognition, social cognition and sensori-motor speed. It has been applied to children, adolescents and adults, including individuals with 22q11.2 deletion [33, 34], where it has been associated with IQ (ICC = 0.57) [36]. The goal of the present project is to examine the pattern of neurocognitive performance on the CNB in a multisite international collaboration and evaluate gene-dosage effects by comparing genomic variants associated with deletion or duplication in 22q11.2 and 16p11.2 loci on multiple domains related to brain function.

## MATERIALS AND METHODS

### Overview

This multisite international collaborative project – “Dissecting the effects of genomic variants on neurobehavioral dimensions in CNVs enriched for neuropsychiatric disorders” – is one of several projects of the G2MH Network. This project includes seven data collection sites, four in North America (Philadelphia, Los Angeles, Montreal, Toronto) and three in Europe (Cardiff, Leuven, Maastricht) and two primary genomic analysis sites (Toronto, San Diego). Here we focus on the prospective data collection of the CNB, describing procedures and results from the current sample. The Institutional Review Boards (IRBs) of the participating institutions approved all study procedures. Informed consent/assent was obtained from each participant or accompanying parent or guardian according to local country guidelines.

### Study Participants

The current sample includes 594 unrelated individuals with IQ > 50, good quality cognitive data, and a CNV at the 22q11.2 or 16p11.2 locus, confirmed by clinical fluorescence *in situ* hybridization (FISH), comparative genomic hybridization, SNP microarray, or multiplex ligation-dependent probe amplification (MLPA) [37]. Participants were recruited from established academic clinical research settings that specialize in the study of rare genetic disorders.

#### Inclusion criteria

1. Participants enrolled are aged ≥ 7 years old. The age range was selected to enable a multifaceted examination of behavioral dimensions and disorders at different settings including home, school, and the community. 2. Able to provide signed informed consent and/or assent, depending on age. 3. Medically stable and able to participate in the evaluation. 4. A sample of blood or saliva is available for DNA extraction for genomic studies.

##### Exclusion Criteria:

Potential participants are excluded if they have any of the following conditions that may affect participation and interpretability of data obtained: 1. Medical or neurological disorders that may substantially affect brain function (e.g., untreatable seizures, significant head trauma, CNS tumor, infection), or visual or auditory limitations (e.g., blindness, deafness). 2. Substance abuse in the past month. 3. Substance dependence not in remission for the past six months. 4. Estimated IQ ≤ 50.

**Figure 1:**
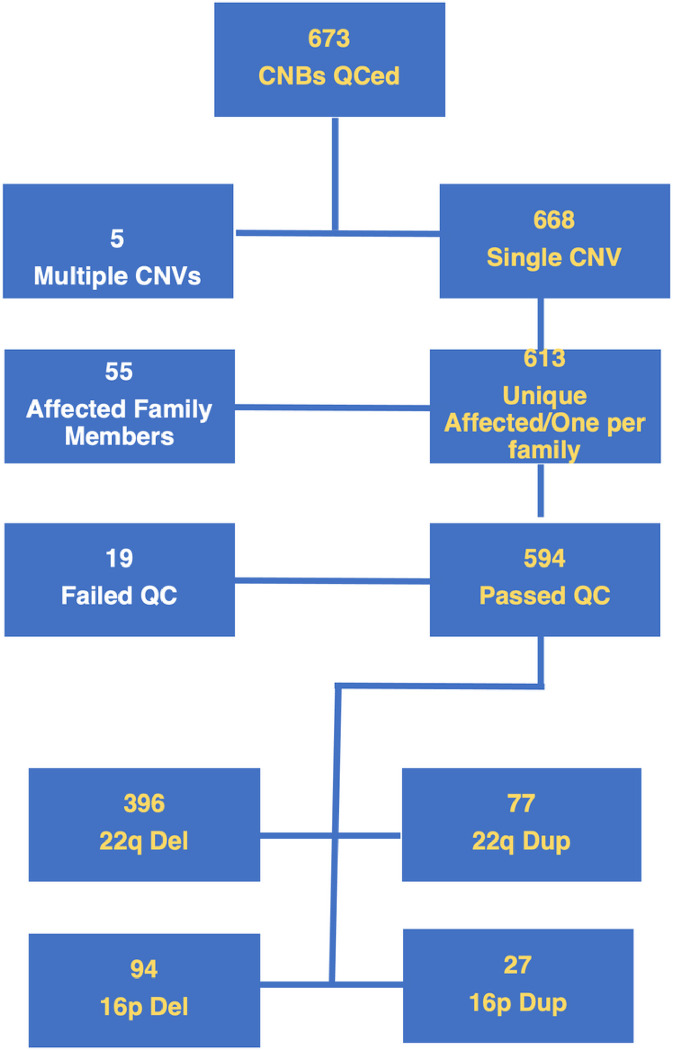
Consort diagram of sample with computerized neurocognitive battery (CNB) data

Figure 1 presents a consort diagram of participants with CNVs at the specified loci who were evaluated with the CNB across all recruitment sites and the reasons for exclusion from the current sample. Notably, we included in the current analysis one proband per family when more than one family member had the specified CNV and excluded individual with additional CNV to the specified loci.

Table 1 presents sample demographic characteristics by 22q11.2 and 16p11.2 loci. As can be seen, the sample for 22q11.2 deletion is the largest, reflecting ongoing collaborations among established centers conducting research with these patients. Females and males are represented across loci and most participants are of European ancestry. Participants’ characteristics were based on self-reports (and/or collateral report), investigators’ observations, and medical records.

### Procedures

#### Neurocognitive assessment.

The Penn CNB [33–35] is a 1-hour computerized battery assessing in the current study five domains across 12 tests: Executive Functions (Abstraction & Mental-Flexibility, Attention, and Working Memory); Episodic Memory (Facial and Spatial Memory); Complex Cognition (Non-Verbal Reasoning and Spatial Processing); Social Cognition (Emotion Identification, Emotion Differentiation, and Age Differentiation); Psychomotor speed (Motor Speed and Sensorimotor Speed). Each test provides measures of both accuracy (number of correct responses) and speed (median time for correct responses), except Psychomotor processing tests that provide only the speed measure. Speed is keyed such that higher values indicate faster performance (RTzscore *−1), and efficiency scores are calculated by averaging the standardized accuracy and speed scores of each test. Notably, we did not include in this study any language tasks (word memory and verbal reasoning) because equating for frequency of words and comparability of linguistic analogies in different languages requires additional steps to ensure validity of tasks.

#### Implementation.

Several steps were taken before starting data collection to ensure that high quality data are obtained in a consistent manner across sites.

#### Translation.

The CNB, established in English, has been translated into multiple languages. For the present study, French and Dutch versions were administered by the Montreal, Toronto, Leuven, and Maastricht sites. The validated translation process included professional translation of the initial version followed by back-translation and discussion with the local teams to assure acceptability, following procedures established in other translations of the CNB.

#### Training.

All clinical coordinators proctoring the CNB were trained with established procedures. These include a training video providing background on testing and describing each test and the required proctor involvement. This video was followed by a quiz requiring a passing grade of 90%. Next, the trainee administered the CNB to an individual and sent the recorded administration to Penn. Feedback was then provided and additional recordings requested if needed for certification.

#### In-Person and Remote Assessment.

At the initiation of the study, all CNBs were administered in-person at the clinical research facilities of each site or at home. With restrictions posed by the COVID-19 pandemic, the Penn CNB team developed and implemented procedures for remote administration. The procedures for remote administration of the CNB followed those of in-person administration [35–36], with certified test administrators proctoring the tests and ensuring a quiet, private setting at participants’ locations. Proctors underwent training on remote assessments, including familiarity with trouble-shooting the remote platform (i.e., Zoom: https://zoom.us). To complete the virtual CNB, administrators provided participants a unique webpage link and participants were instructed to share their screen with the proctor, so that their performance can be monitored in real time. Through the screen share, the proctor dictated all instructions and observed the participant’s responses for each task. For younger individuals, a parent was present before the assessment started and remained available if needed. No differences were found between in-person and remote administration modes in studies using the CNB (manuscript in submission).

#### Data quality assurance.

This step involved a rigorous validation process that used three methods. First, validation codes from the trained test assessors proctoring the CNB indicated when the quality of data was unusable (e.g., participant not engaged or stopped performing task). Second, Penn CNB auto-validation rules were implemented. These are hard-coded, test-specific rules developed to protect against poor data quality that can result from several factors (e.g., unreasonably short response times, unusual repetition of same-key response, unmotivated responding, intentional poor performance, fatigue, etc.). Recently, a third approach that uses data-driven performance validity metrics was also calculated for all tests except for abstraction and mental flexibility and tapping speed tests. Data were excluded from analyses if the test was flagged on two or more of the above methods without removing the entire session, such that an individual could have data for some tests but not others. If a participant was missing a test, imputation using the random forest procedure was performed before averaging accuracy or speed [38]. Of the 613 probands with the identified loci and CNB data, 3.1% were excluded from analysis due to failing QA. This proportion is similar to other studies that have used the CNB. Healthy controls who were administered the CNB at Penn under the same procedures as the CNVs carriers provided normative data across the age range and were balanced for sex and ethnicity. They were medically and psychiatrically assessed and were free of disorders that may impact cognitive performance [33–35].

### Statistical Analysis

The accuracy and speed scores on the tests were z-transformed using the normative means and standard deviations from the matched sample of healthy controls. These z-scores served as the dependent measures in a series of Mixed Model Repeated Measures (MMRM) analyses with Locus (22q11.2 vs 16p11.2) and Deletion vs Duplication as between-group factors and Test as the repeated-measures (within group) factor. The Test vector included Abstraction and mental flexibility, Attention, Working memory, Face memory, Shape memory, Nonverbal (Matrix) reasoning, Spatial processing, Emotion identification, Emotion intensity differentiation, Age differentiation; Sensorimotor and motor speed were added when Speed was the dependent measure. Age and sex were entered as covariates. Site effects were further examined by adding site as a grouping factor within the same MMRM design, separated by loci. We also examined correlations between the CNB performance and IQ measures available in the database using the Pearson product moment correlation. We recognize that sex is an important biological variable, but the sample size is insufficiently powered across groups to examine group x sex interactions.

## RESULTS

### Locus Effects

Results of the Locus (22q11.2, 16p11.2) x Deletion-Duplication x Test Mixed Model analysis on the Accuracy and Speed data are presented in Table 2. As can be seen, for both Accuracy and Speed there was a significant Locus x Deletion-Duplication and a Locus x Deletion-Duplication x Test interaction (all p < 0.01).

**Figure 2:**
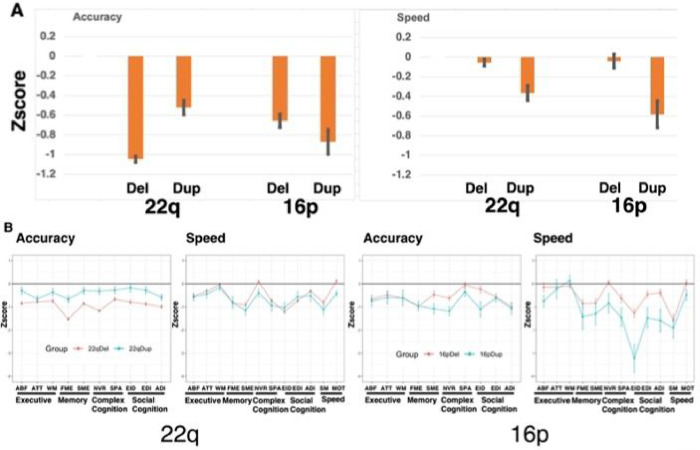
Neurocognitive performance of the groups by Locus and deletion vs duplication status. Top panels: Means (+/−SEM) of performance of the four groups in Accuracy (left panel) and Speed (right panel) averaged across tests. Bottom panels: Neurocognitive profiles of the four groups showing means (+/−SEM) of performance for Accuracy and Speed.^a^ ^a^ Del=group with deletions, Dup=group with duplications, Del=deletion, Dup=duplication, ABF=Abstraction and Mental Flexibility, ATT=Attention, WM=Working Memory, FME=Face Memory, SME=Spatial (shape) Memory, NVR=Nonverbal (matrix) Reasoning, SPA=Spatial Processing, EID=Emotion Identification, EDI=Emotion intensity Differentiation, ADI=Age differentiation, SM=Sensorimotor speed, MOT=Motor Speed.

The Locus x Deletion-Duplication interactions and the Locus x Deletion-Duplication x Test interactions are shown in Fig. 2.

As can be seen in Fig. 2 (top panel, left bars), the 22q11.2 deletion group was more impaired in average performance Accuracy than the 22q11.2 duplication group, while the reverse was the case for the 16p11.2 locus, where the duplication group was more impaired than the deletion group. The interaction results for Speed (2 top right panel) indicates that for both loci, 22q11.2 and 16p11.2, duplications were associated with greater deficits than deletions.

Figure 2, bottom panels, shows the group effects for Accuracy and Speed by test. For the 22q11.2 locus, the deletion group was more impaired than the duplication group on Accuracy across tests, but especially in face memory and non-verbal reasoning. Speed was comparable for the two groups across tests, perhaps indicating a speed-accuracy tradeoff for the 22q11.2 duplication group. In contrast to the 22q11.2 locus groups, the 16p11.2 duplication group showed overall greater impairment than the 16p11.2 deletion group. This effect was evident for Accuracy in some domains and for Speed across nearly all domains. Particularly notable for the 16p11.2 duplication group was reduced speed of emotion identification and other social cognition and complex cognition domains. Thus, across Accuracy and Speed, the 16p11.2 duplication group was most impaired on non-verbal reasoning and social cognition.

### Association with IQ

**Figure 3:**
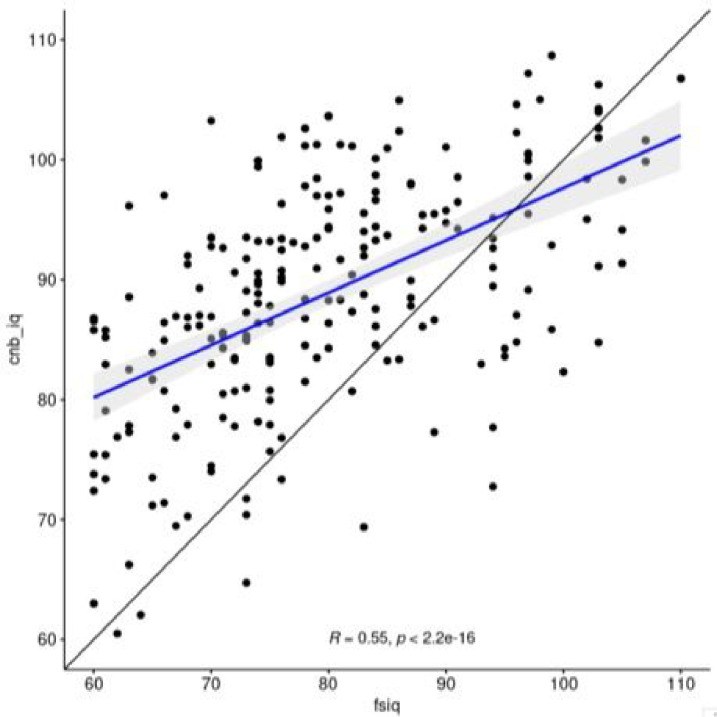
Scatterplot showing the association between full-scale IQ in records and the average performance accuracy on the CNB.^a^ ^a^fsiq=Full-scale IQ score from records, cnb_iq=computerized testing-based IQ estimate based on averaging accuracy across CNB tests.

To allow better bridging of our CNB findings with available literature where an IQ measure has most frequently been used to assess cognitive capacity, we evaluated the association between the CNB estimate of overall accuracy, defined as the average accuracy z-scores across tests, and IQ data available from the participants’ health or research records indicating Wechsler scales scores (WAIS-3,4;WASI-1,2: WISC-3–5; WPPSI-4). The association between the two measures (Fig. 3), is moderate, r(369) = 0.57, p < 0.001, with the computerized estimates consistently higher than the paper-and-pencil based measures, especially at the lower ranges of performance, as indicated by the majority of observations above the identity line. As can be seen all participants had IQ above 60. Notably, the interval between IQ provided in clinical records (younger) and the CNB assessment averaged 3.40(± 5.15SD) years. The partial correlation between the measures was 0.60 after partialling out the interval between the measures.

### Site Effects

We also tested for site effects using the same Mixed Model design and adding Site as a factor. Analyses evaluating site effects are presented in Supplementary Tables S1 (for 22q11.2 locus) and S2 (for 16p11.2 locus) and indicated no significant effects or interactions.

## DISCUSSION

The adverse effects of CNVs on cognitive performance have been documented in the literature in studies that used a range of different measures. Most commonly an “intelligent quotient” (IQ) has been used based on standardized clinical tests (e.g., Wechsler scales). More extensive neurocognitive assessments have been reported in clinical and research samples that included examination of both deletions and duplications of CNVs such as 22q11.2 and 16p11.2 [31, 32]. However, currently published reports do not permit comparing locus effects on a range of neurocognitive domains with the same detailed measurements in both deletion and duplication. The present study addresses this gap by reporting results of a comprehensive neurocognitive evaluation of a large multi-site sample of individuals with deletion or duplications at the 22q11.2 or 16p11.2 loci. The computerized neurocognitive battery (CNB) is an extensively validated and efficient instrument that is based on cognitive neuroscience and provides information on accuracy and speed of performance in neurocognitive domains related to brain systems [39]. The data in the current study were collected prospectively following rigorous training and quality assurance procedures. The administration was well tolerated by participants across sites, as evident in the low percentage of quality control rejections (3.1%), and no site-effects were observed across North America and Europe.

The overall results indicate that whereas deletions are more deleterious than duplications at the 22q11.2 locus, the opposite effects were seen at the 16p11.2 locus, where duplications were more deleterious than deletions. Our findings for the 22q11.2 locus are consistent with earlier studies showing that the cognition effects of the deletion are more deleterious than those of the duplication [20]. The literature on 16p11.2 is less consistent [31, 32] and our study clarifies that overall for this locus the deletion is less deleterious than the duplication with respect to cognitive performance. Notably, the two CNVs associated with schizophrenia risk, 22q11.2 deletion and 16p11.2 duplication [40], show greater deficits than the other groups on Face Memory, Spatial Memory, Nonverbal Reasoning and Emotion Identification.

The computerized format allows separate evaluations of accuracy and speed of performance, and our analysis revealed further specific differences among the groups. At both 22q11.2 and 16p11.2 loci, deletions and duplications were associated with reduced accuracy, whereas duplications were also associated with reduced speed. The pattern of performance across domains also differed among the four groups. For the 22q11.2 loci, deletion affected accuracy across nearly all domains, while duplication was associated with milder impairment in accuracy albeit with slower speed. For the 16p11.2 loci, deletion and duplication had the same effect on accuracy of performance on some domains - executive functions, face memory, spatial processing, emotion and age differentiation - but those with a duplication performed more poorly on spatial memory, nonverbal reasoning and emotion identification. Notably, the effects of 16p11.2 loci were most pronounced for speed especially for complex cognition and social cognition. These results can inform common and differing mechanisms through which such CNVs impact cognition and brain function. Such investigations could link individual differences in performance with brain structural and functional parameters to allow individual characterization. For example, a multimodal MRI study of 22q11.2 deletion syndrome showed that primary visual processing and insular function were relatively intact in individuals with the deletion, while motor feedback, face processing, and emotional memory processes were more impaired compared to controls. Such approaches may help inform potential intervention targets and enhance the specificity of neuroimaging and electrophysiological indices related to cognitive dysfunction [41]. Furthermore, mechanistic insights on the neurobehavioral deficits can benefit from preclinical work, which has identified 22q11.2 and 16p11.2 genes that contribute to the accuracy and speed of social and cognitive functions in mice (e.g., 42–44).

As most previous studies have examined IQ as a measure of cognition, we related global performance on the computerized neurocognitive battery to IQ measures available in clinical and research records and/or assessed across the sites. The correlation between these measures was moderate (r = 0.57) in this study, consistent with an earlier study reporting a correlation of 0.57 between IQ and CNB average accuracy or 22q11.2 deletion alone [36]. The association between IQ and average CNB performance is probably attenuated by the time difference between the assessment, as suggested by the increase in association to 0.60 after partialling out this interval. It is likely further attenuated by the various instruments used for measuring IQ. Notably, the CNB is based on functional neuroimaging and includes domains that are not assessed in IQ tests.

### Limitations

While we report the results of a large collaborative study for rare CNVs, the sample size across the loci varies. Most participants are of European ancestry, reflecting location of sites and perhaps these CNVs being underdiagnosed in other ancestries. This issue needs further investigation. Differential ascertainment for variants is another potential limitation. For example, individuals with 22q11.2 deletions are more likely to be referred for testing for physical issues such as congenital heart defects, whereas those with 22q11.2 duplications are more likely to be referred for developmental reasons. The healthy controls for standardizing performance were from the University of Pennsylvania normative database, as collection of normative controls was not part of the funded study. A more rigorous approach would have entailed collection of normative samples at each site. However, there is evidence that normative data from the University of Pennsylvania are comparable to other settings [6, 36]. Notably, age was a significant covariate even though the CNB measures were age normed. This effect indicates that trajectories of people with CNVs across age differ from those of typically developing individuals. However, the age range across the loci was broad and sample sizes limited systematic examination of age bins, which can be performed in the future with larger samples. A more comprehensive analysis of IQ data is beyond the scope of this report. We are not reporting here on breakpoints in the CNV loci, as this information will be part of the whole-genome sequencing that will become available at the conclusion of the study. Similarly, the association of neurocognitive performance with neuropsychiatric disorders, which are common in these CNVs, medications, and other medical conditions will be examined as part of the next phase of the study.

Notwithstanding these limitations, our study demonstrates that efficient prospective measures can help identify differential CNV effects on neurocognition. Our approach offers a unique opportunity to characterize the functional consequences of genetic mutations highly penetrant for multiple neuropsychiatric disorders. They have implications for genotype-phenotype relationships in psychiatry. Different psychiatric risk variants result in different cognitive profiles and trajectories and may represent different pathways to psychiatric outcomes. Examining cognitive endophenotypes provides a step further in understanding the route from genomic risk to psychiatric outcomes. Future studies could further elucidate unique and common features associated with CNVs, pointing to mechanistic links between genomic variations and their phenomic manifestations.
